# Tophaceous Gout at the Popliteal Sulcus

**DOI:** 10.5334/jbsr.3523

**Published:** 2024-03-26

**Authors:** Vincent Alberts, Frederiek Laloo, Benjamin Leenknegt

**Affiliations:** 1 Ziekenhuis Oost-Limburg, Genk, Belgium; 2 AZ Sint-Lucas, Ghent, Belgium; 3 AZ Sint-Lucas, Ghent, Belgium

**Keywords:** Gout, Knee, Tophaceous Gout, Popliteal sulcus, Popliteal Tendon, X-ray, MRI, Ultrasound

## Abstract

*Teaching point:* Popliteal sulcus erosion with soft tissue mass: when in doubt, think of gout.

## Case History

An 81-year-old patient, admitted to our hospital after a neurologic insult, complained about a painful right knee. During clinical examination, a swollen, painful right knee was noted. In particular, palpation at the medial rim of the medial tibial plateau induced an expression of pain. There was no relevant medical history.

## Images

**Figure 1:**
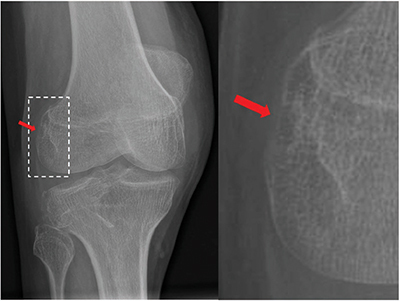
Radiography of the right knee demonstrating an erosion of the popliteal sulcus (red arrow).

Radiography of the knee demonstrated a frank joint effusion and erosion of the popliteal sulcus (Figure 1, red arrow).

Ultrasound confirmed the intra-articular effusion and demonstrated synovial hyperplasia.

**Figure 2:**
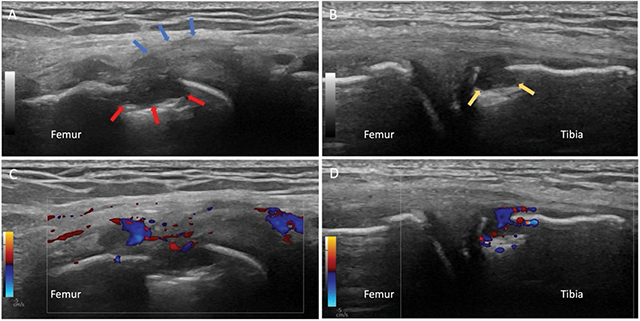
Ultrasound of the right knee demonstrating a thickened popliteal tendon and erosion of the the popliteal sulcus (**A** and **C**). A second erosion was shown on the medial rim of the medial tibial plateau (**B** and **D**).

On the lateral side, the popliteal tendon—which lies deep in the lateral collateral ligament—was diffusely thickened (blue arrows, Figure 2A). Just adjacent to this tendon insertion, erosion is also observed (red arrows, Figure 2A). A hypoechoic, soft tissue nodule with power doppler activity fills the erosion (Figure 2C).

At the painful spot, on the medial rim of the medial tibial plateau, a second erosion is seen on ultrasound (Figure 2B, yellow arrows). Again, we recognize a hypoechoic and hyperemic tophus within the erosion (Figure 2D). Both erosions also demonstrate small overhanging edges not visible on radiography.

Gouty arthritis of the right knee was suggested, and an ultrasound-guided knee aspiration was performed. The presence of monosodium urate crystals confirmed our hypothesis.

**Figure 3:**
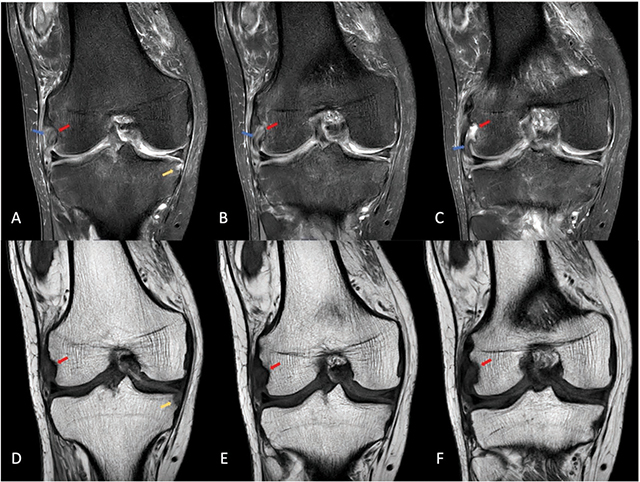
MRI of the right knee demonstrating a diffusely thickened, hyperintense popliteal tendon on proton density-weighted images (blue arrows), an erosion of the popliteal sulcus (red arrows) and an erosion at the medial rim of the medial tibial plateau (yellow arrow).

An additional magnetic resonance imaging (MRI) was requested to explore the extent of the disease (Figure 3).

On MRI, a complex joint effusion with synovial proliferation is demonstrated. The popliteus tendon (blue arrows, Figure 3) is thickened and diffusely hyperintense at its insertion on the proton density-weighted images. The erosions at the popliteal sulcus (red arrows, Figure 3) and on the medial rim of the medial tibial plateau are also seen (yellow arrow, Figure 3).

## Comments

Tophaceous gout is usually a chronic manifestation of the disease. The knee is the third most involved large joint, after the foot and the ankle.

Diagnosis is typically based on laboratory test results (elevated serum urate levels), clinical history, physical exam (swollen, painful joint), imaging, and joint effusion analysis.

The imaging findings of tophaceous gout in the knee are usually nonspecific and may mimic inflammatory arthritis, pigmented villonodular synovitis (PVNS) or amyloidosis.

Tophi can emerge anywhere around the joint, often in multiple locations. The origin of the popliteus tendon is a common location of knee joint gout. In a study group of 30 patients with tophaceous gout, there was involvement at the popliteal sulcus in 78% (25) and involvement at the medial rim of the medial condyle in 19% (6) of patients [1].
